# Surgical approach selection and perioperative-pathological characteristics in endometrial cancer: a retrospective observational study

**DOI:** 10.3389/fmed.2026.1870788

**Published:** 2026-06-17

**Authors:** Ali Deniz Erkmen, Sedat Akgöl

**Affiliations:** Division of Gynecologic Oncology, Gazi Yaşargil Training and Research Hospital, Diyarbakır, Türkiye

**Keywords:** endometrial cancer, laparoscopy, laparotomy, lymph node, V-NOTES

## Abstract

**Objective:**

To evaluate real-world surgical selection patterns and associated perioperative, pathological, and follow-up findings in patients with endometrial cancer undergoing laparotomy, laparoscopy, or V-NOTES.

**Materials and methods:**

This retrospective study included 151 patients who underwent surgical treatment for endometrial cancer between 2018 and 2025 at a tertiary referral center. One hundred forty-seven patients were analyzed, including 84 who underwent laparotomy, 20 laparoscopy, and 43V-NOTES. Demographic characteristics, preoperative findings, operative outcomes, final pathology, adjuvant treatment, and follow-up data were compared. Continuous variables were analyzed using the Kruskal–Wallis test, and categorical variables using the chi-square or Fisher’s exact test. A multivariable logistic regression model was constructed to identify factors associated with adjuvant treatment requirement.

**Results:**

Significant differences in baseline characteristics were observed across surgical groups. Patients in the laparotomy group were older, more frequently postmenopausal, and had higher-risk disease features, including deeper myometrial invasion, higher tumor grade, and a greater proportion of high-risk histological subtypes (all *p* < 0.05). Operative time and estimated blood loss were significantly higher in the laparotomy group (both *p* < 0.001). Lymph node assessment strategies differed markedly, with systematic pelvic and para-aortic lymphadenectomy predominantly performed in the laparotomy group, whereas sentinel lymph node dissection (SLND) was more common in minimally invasive approaches (*p* < 0.001). Adverse pathological features, including lymphovascular space invasion (LVSI) and advanced FIGO stage, were more frequently observed in the laparotomy group (*p* = 0.002 and *p* ≤ 0.010, respectively). Adjuvant treatment was administered more frequently after laparotomy (*p* < 0.001). However, in multivariable analysis, surgical approach was not independently associated with adjuvant treatment requirement, whereas postoperative tumor grade (OR = 5.78, *p* < 0.001) and deep or serosal myometrial invasion (OR = 35.23, *p* < 0.001) remained significant predictors. Median follow-up duration among patients with available follow-up data was 12.4 months (IQR: 7.4–15.4 months).

**Conclusion:**

The observed differences in perioperative, pathological, and treatment findings among surgical approaches appear to reflect underlying variations in patient and tumor characteristics rather than the intrinsic effects of the surgical techniques themselves. In routine clinical practice, minimally invasive approaches are more commonly utilized in lower-risk patients, whereas laparotomy is more frequently performed in patients with advanced or high-risk disease.

## 1 Introduction

Endometrial cancer is the most common gynecologic malignancy in developed countries, and its incidence continues to increase worldwide, largely driven by aging populations, obesity, diabetes, and other metabolic risk factors (1, 2). Although many patients are diagnosed at an early stage due to abnormal uterine bleeding, a substantial proportion present with high-risk pathological features such as deep myometrial invasion, lymphovascular space invasion (LVSI), non-endometrioid histology, and advanced-stage disease, all of which are strongly associated with recurrence risk and survival outcomes (3, 4).

Surgical staging remains the cornerstone of treatment in endometrial cancer. Standard management typically includes total hysterectomy with bilateral salpingo-oophorectomy, with lymph node assessment performed according to individual risk stratification (5). Contemporary clinical guidelines from the National Comprehensive Cancer Network (NCCN) (6), European Society of Gynaecological Oncology (7), and Society of Gynecologic Oncology support the use of minimally invasive surgery (MIS) as the preferred approach in patients with apparent uterine-confined disease, given its association with reduced perioperative morbidity and comparable oncologic outcomes in appropriately selected cases (8–10).

Over the past two decades, minimally invasive approaches, particularly conventional laparoscopy, have increasingly replaced laparotomy in the management of early-stage endometrial cancer (11). Large randomized trials, including the GOG-LAP2 and LACE trials, have demonstrated that laparoscopic surgery provides comparable oncologic outcomes to open surgery while reducing perioperative morbidity (12, 13). More recently, vaginal natural orifice transluminal endoscopic surgery (V-NOTES) has emerged as a distinct minimally invasive approach combining vaginal access with endoscopic visualization. Increasing interest in V-NOTES has paralleled the broader adoption of sentinel lymph node–based staging strategies, particularly in patients with presumed uterine-confined or low-risk disease (14–16). Unlike conventional laparoscopy, V-NOTES differs in surgical access route and retroperitoneal staging workflow, which may influence operative selection and lymph node assessment strategies in clinical practice (17, 18). Although early studies suggest that V-NOTES may be feasible and safe in carefully selected patients, evidence regarding its oncologic adequacy and applicability in higher-risk disease remains limited (19–22).

Despite the growing use of minimally invasive techniques, interpretation of comparative surgical findings in observational studies remains challenging because surgical allocation is strongly influenced by patient characteristics, tumor biology, imaging findings, and anticipated procedural complexity. In routine practice, laparotomy is more commonly performed in patients with advanced-stage disease, high-grade tumors, or suspected nodal involvement, whereas minimally invasive approaches are preferentially utilized in lower-risk, uterine-confined cases (23–25). This non-random allocation introduces substantial confounding and limits direct attribution of perioperative or oncologic differences to surgical approach alone.

Furthermore, the increasing adoption of sentinel lymph node mapping has added another layer of complexity to surgical decision-making (26). Differences in lymph node assessment strategies may independently influence operative time, blood loss, and postoperative recovery, thereby confounding comparisons between surgical techniques (17, 27). In addition, direct comparisons between laparoscopy and V-NOTES remain relatively limited, particularly with respect to final pathological characteristics, stage distribution, and adjuvant treatment patterns in real-world clinical settings (28, 29).

Therefore, rather than aiming to establish causal superiority of one surgical approach over another, the present study was designed to evaluate real-world surgical selection patterns and associated perioperative, pathological, and follow-up outcomes in patients with endometrial cancer undergoing laparotomy, laparoscopy, or V-NOTES. By examining how these approaches are applied across heterogeneous patient populations, this study aims to provide a clinically contextualized understanding of contemporary surgical practice in endometrial cancer management.

## 2 Materials and methods

### 2.1 Study design and patient selection

**Figure 1:**
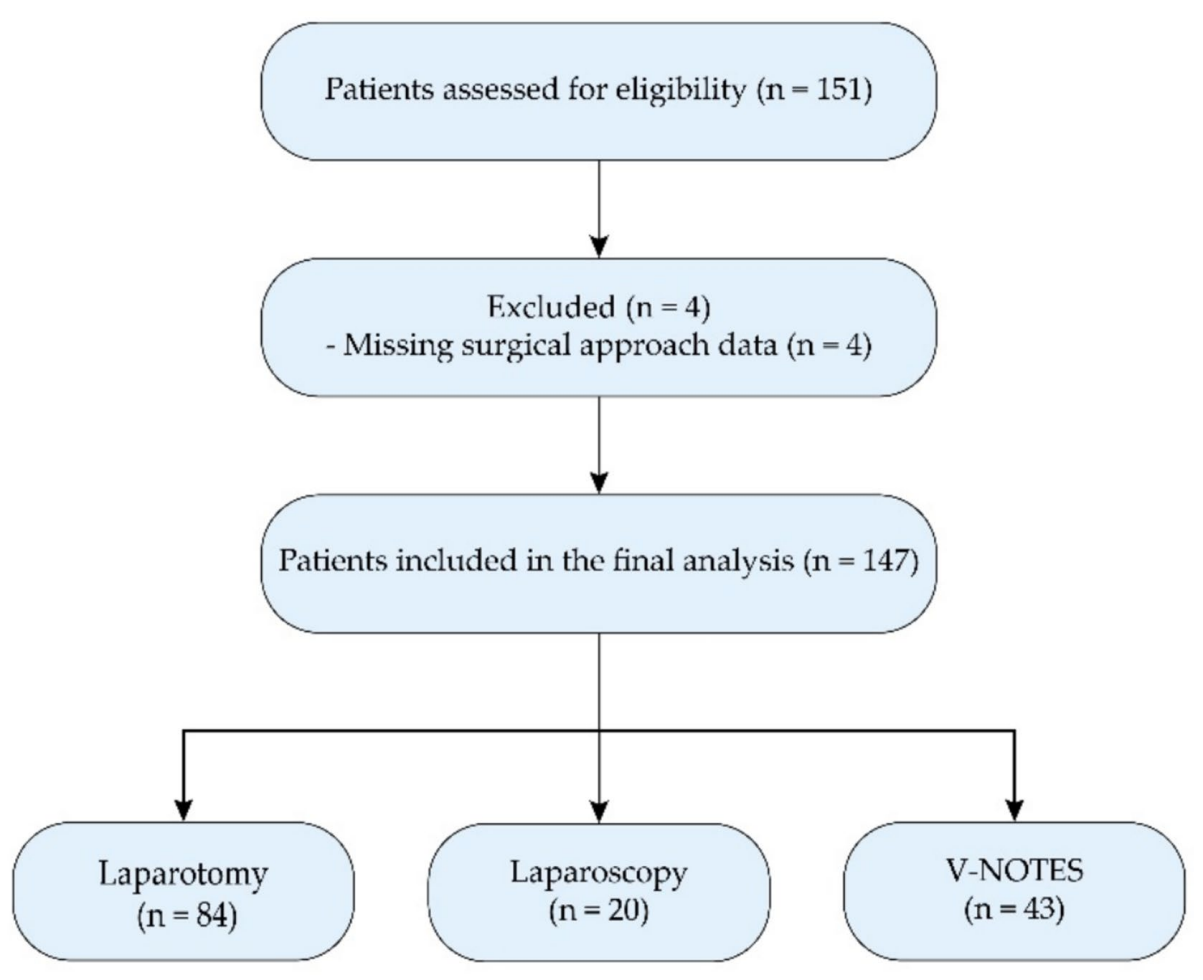
Flow diagram of patient selection and study population. A total of 151 patients were identified; 4 were excluded due to missing surgical approach data. The final cohort included 147 patients: laparotomy (*n* = 84), laparoscopy (*n* = 20), and V-NOTES (*n* = 43).

This study was designed as a retrospective observational cohort study conducted at a tertiary referral center. The primary objective of the study was to evaluate real-world surgical selection patterns among patients undergoing laparotomy, laparoscopy, or V-NOTES for endometrial cancer. Secondary exploratory objectives included comparison of associated perioperative, pathological, adjuvant treatment, and follow-up findings across surgical groups. All patients who underwent surgical treatment for endometrial cancer between January 2018 and December 2025 were identified from institutional electronic medical records. Patients were included if they underwent laparotomy, conventional laparoscopy, or vaginal natural orifice transluminal endoscopic surgery (V-NOTES). Patients with missing surgical approach data, incomplete pathological records, or unavailable follow-up information were excluded from the comparative analyses. A total of 151 patients were initially identified. After excluding four patients with missing surgical approach data, 147 patients were included in the final analysis. Patients were categorized into three groups according to surgical approach: laparotomy, laparoscopy, and V-NOTES (Figure 1). The four excluded cases had incomplete archived operative records in which the surgical route could not be reliably verified; because these patients represented only 2.6% of the initially identified cohort, no formal sensitivity analysis was performed.

Given that the choice of surgical approach was determined based on clinical and tumor-related factors in routine practice, baseline differences between groups were expected, and the analyses were therefore interpreted within the context of non-randomized group allocation. All procedures were performed by gynecologic oncologists experienced in both minimally invasive and open surgical techniques; however, surgeon-specific procedural preferences and differences in experience levels could not be standardized due to the retrospective design. Due to the retrospective nature of the study, certain secondary variables were not available for all patients. Therefore, denominators may vary across variables, and percentages were calculated based on available data. The study was conducted in accordance with the principles of the Declaration of Helsinki and was approved by the local ethics committee (Approval date: 19.12.2025; approval number: 805). In addition to descriptive comparisons among surgical groups, the study also aimed to explore which clinicopathological factors were associated with surgical allocation patterns and postoperative adjuvant treatment decisions in routine clinical practice.

### 2.2 Data collection

Demographic, clinical, operative, pathological, and follow-up data were retrospectively collected from electronic medical records, operative reports, imaging findings, and pathology reports. The following variables were recorded:

- Demographic and clinical variables: age at diagnosis, menopausal status, parity, smoking status, and presenting symptoms
- Preoperative findings: transvaginal ultrasonographic endometrial thickness, tumor size, myometrial invasion status, PET findings (SUVmax and lesion size, when available), preoperative histopathological diagnosis, and tumor grade
- Operative variables: surgical approach, operation time, estimated blood loss, transfusion requirement, intraoperative complications, and lymph node assessment method
- Pathological variables: final histopathological diagnosis, postoperative tumor grade, depth of myometrial invasion, cervical stromal invasion, uterine serosal involvement, adnexal involvement, surgical margin status, LVSI, and lymph node metastasis
- Staging and treatment variables: FIGO 2023 stage, adjuvant treatment status and modality
- Follow-up variables: recurrence status, imaging findings during follow-up, and tumor marker levels (CA-125, CA 19–9, and CEA)

Surgical procedures were not standardized due to the retrospective nature of the study. All patients underwent total hysterectomy with bilateral salpingo-oophorectomy; however, the extent of lymph node assessment and surgical staging varied according to intraoperative findings, preoperative risk assessment, and institutional practice patterns. Lymph node assessment included sentinel lymph node dissection (SLND), pelvic lymph node dissection, para-aortic lymph node dissection, or combined procedures, depending on clinical indication. Patients with suspected advanced disease, high-grade tumors, or radiologically suspicious lymph nodes were more likely to undergo more extensive lymphadenectomy and open surgery. The choice between laparotomy and minimally invasive approaches, as well as between laparoscopy and V-NOTES, was based on a combination of clinical and technical factors, including tumor risk profile, suspected disease extent, uterine size, vaginal accessibility, history of prior abdominal surgery, and surgeon experience with specific techniques. Transumbilical single-port laparoscopy was not routinely utilized at our institution during the study period.

All procedures were performed by gynecologic oncologists experienced in both minimally invasive and open surgical techniques; however, surgeon-specific procedural preferences and differences in experience levels could not be standardized due to the retrospective design. In the present cohort, para-aortic lymph node dissection was not routinely performed through the V-NOTES approach because of technical and patient-selection considerations. No intraoperative conversions from V-NOTES to laparotomy or conventional laparoscopy were identified in the available operative records.

### 2.3 Pathological evaluation

All surgical specimens were evaluated by experienced gynecologic pathologists at the same institution. Histopathological assessment included tumor subtype, tumor grade, depth of myometrial invasion, cervical stromal invasion, uterine serosal involvement, adnexal involvement, surgical margin status, LVSI, and lymph node status. Tumors were classified according to standard histopathological criteria. For analytical purposes, endometrioid carcinoma included low-grade and high-grade endometrioid subtypes, whereas tumors with serous, clear cell, carcinosarcoma, or mixed histological features were considered within the high-risk histological group. Myometrial invasion was categorized as:

- no invasion
- <50% invasion
- ≥50% invasion
- serosal involvement

Tumor grade was classified as grade 1, grade 2, or grade 3 based on the final pathological report. Tumors were staged according to the FIGO 2023 classification system (30). Although propensity score–based methods could theoretically reduce selection bias, their application was not feasible in this study due to the relatively small sample size, particularly in the laparoscopy group, and the substantial imbalance between groups, which would likely result in significant loss of statistical power and model instability.

### 2.4 Follow-up and adjuvant treatment

Postoperative management decisions, including the use of adjuvant treatment, were made by a multidisciplinary gynecologic oncology team based on established clinical and pathological risk factors. These factors included tumor grade, histological subtype, depth of myometrial invasion, LVSI, lymph node involvement, and FIGO stage. Adjuvant treatment modalities included chemotherapy, radiotherapy, chemoradiotherapy, brachytherapy, or combined treatment approaches. For analytical purposes, adjuvant treatment was evaluated as a binary variable (administered vs. not administered). Patients who refused treatment were recorded separately and were excluded from binary comparisons of adjuvant treatment.

Follow-up data were obtained from outpatient clinic records and included recurrence status, imaging findings, and tumor marker levels (CA-125, CA 19–9, and CEA), when available. Follow-up duration was calculated as the interval between the date of diagnosis and the last available follow-up visit recorded in the institutional database. Recurrence was defined based on radiological evidence, pathological confirmation, or documented clinical progression. Because recurrence-date and survival-event data were not consistently available, formal time-to-event analyses such as disease-free survival (DFS) and overall survival (OS) could not be performed. Accordingly, follow-up analyses were limited to descriptive comparisons among patients with available follow-up data, and patients without follow-up information were excluded from recurrence analyses. Given the limited sample size relative to the number of predictors included in the multivariable model, the results should be interpreted cautiously because of the potential risk of model overfitting.

### 2.5 Statistical analysis

Statistical analyses were performed using IBM SPSS Statistics software (Version 25.0; IBM Corp., Armonk, NY, USA). The distribution of continuous variables was assessed using the Shapiro–Wilk test. Because most continuous variables were not normally distributed, data were presented as median and interquartile range (IQR). Categorical variables were summarized as number (n) and percentage (%). Comparisons among the three surgical groups were performed using the Kruskal–Wallis test for continuous variables and the chi-square test or Fisher’s exact test, as appropriate, for categorical variables. For variables demonstrating significant omnibus differences, post-hoc pairwise comparisons with multiple-comparison adjustment were additionally performed and are presented in Supplementary Table S1. Because missingness was primarily related to incomplete retrospective clinical documentation rather than predefined study procedures, complete-case analysis was used for multivariable regression modeling. The extent of missing data for key clinicopathological, treatment, and follow-up variables is summarized in Supplementary Table S2. Given the retrospective design and non-randomized allocation of surgical approach, univariate comparisons were interpreted within the context of substantial baseline imbalance between groups. To further evaluate perioperative findings, separate multivariable linear regression models were constructed for operative time and estimated blood loss. Surgical approach and lymph node dissection type were included simultaneously in the regression models. Robust standard errors were used because of heteroscedasticity and non-normal distribution observed in perioperative outcome variables.

Multivariable logistic regression analysis was additionally performed to explore factors independently associated with adjuvant treatment requirement after adjustment for clinically relevant pathological variables. Adjuvant treatment status (yes/no) was used as the dependent variable. Surgical approach was intentionally retained in the multivariable model because the study specifically aimed to evaluate whether observed differences in adjuvant treatment patterns persisted after adjustment for major pathological risk factors. Variables included in the model were selected based on clinical relevance and univariate analysis findings and included age, surgical approach, postoperative tumor grade, depth of myometrial invasion, and LVSI status. Results were reported as odds ratios (OR) with 95% confidence intervals (CI). Only patients with complete data for all variables included in the model were analyzed. Patients who refused adjuvant treatment and those with missing data were excluded from the regression analysis. Because recurrence-date and survival-event data were not consistently available, formal survival or time-to-event analyses could not be performed. Therefore, recurrence-related findings were analyzed descriptively. A two-sided *p*-value of <0.05 was considered statistically significant.

## 3 Results

### 3.1 Baseline demographic and preoperative characteristics

A total of 151 patients were initially identified in the dataset. After excluding four patients with missing surgical approach data, a total of 147 patients were included in the comparative analyses. Of these, 84 patients (57.1%) underwent laparotomy, 20 patients (13.6%) underwent laparoscopy, and 43 patients (29.3%) underwent V-NOTES. Baseline demographic and preoperative characteristics are summarized in Table 1. There was a statistically significant difference in age distribution among the groups, with patients in the V-NOTES group being younger than those in the laparotomy and laparoscopy groups (*p* < 0.001). Similarly, menopausal status differed significantly across groups (*p* = 0.023), with a higher proportion of postmenopausal patients in the laparotomy group.

**Table 1:** Baseline demographic and preoperative characteristics according to surgical approach.

Variable		Laparotomy *n* = 84	Laparoscopy *n* = 20	V-NOTES *n* = 43	*p*
Age, years		61.0 (53.0–70.0)	59.5 (50.8–65.2)	53.0 (47.0–60.0)	<0.001
Menopausal status	Premenopausal	5 (6.0%)	2 (10.0%)	6 (14.3%)	0.023
	Postmenopausal	60 (71.4%)	12 (60.0%)	17 (40.5%)	
	Perimenopausal	19 (22.6%)	6 (30.0%)	19 (45.2%)	
Parity		5.0 (3.0–7.0)	3.0 (1.5–4.5)	4.0 (3.0–6.0)	0.071
Smoking		8 (9.5%)	1 (5.0%)	7 (16.3%)	0.339
Socioeconomic status	Good	1 (1.2%)	1 (5.0%)	1 (2.3%)	0.431
	Moderate	75 (89.3%)	19 (95.0%)	40 (93.0%)	
	Poor	8 (9.5%)	0	2 (4.7%)	
Presenting symptom	Postmenopausal bleeding	67 (79.8%)	13 (65.0%)	17 (39.5%)	<0.001
	Premenopausal bleeding	16 (19.0%)	6 (30.0%)	23 (53.5%)	
	Postcoital bleeding	0	0	3 (7.0%)	
	Other/no symptom	1 (1.2%)	1 (5.0%)	0	
TV-USG endometrial thickness, mm		30.0 (18.2–44.8)	22.0 (11.5–35.0)	20.0 (15.0–27.0)	0.029
TVUS mass size, cm		3.0 (1.8–5.0)	2.5 (1.9–4.5)	2.2 (1.7–3.0)	0.058
TVUS myometrial invasion	No invasion	6 (9.2%)	4 (30.8%)	9 (28.1%)	0.033
	<50% invasion	15 (23.1%)	0	5 (15.6%)	
	≥50% invasion	38 (58.5%)	9 (69.2%)	18 (56.2%)	
	Serosal invasion	6 (9.2%)	0	0	
PET SUVmax		17.7 (11.2–20.6)	13.2 (7.6–16.5)	13.5 (10.0–17.5)	0.078
PET lesion size, mm		34.0 (19.9–45.0)	25.5 (17.0–41.2)	25.0 (18.0–30.0)	0.101
Preoperative pathology	EIN / hyperplasia	1 (1.2%)	5 (25.0%)	11 (25.6%)	<0.001
	Endometrioid carcinoma	60 (74.1%)	15 (75.0%)	32 (74.4%)	
	Serous carcinoma	9 (11.1%)	0	0	
	Clear cell carcinoma	1 (1.2%)	0	0	
	Sarcoma/carcinosarcoma/mixed high-risk	7 (8.6%)	0	0	
	Adenosquamous carcinoma	3 (3.7%)	0	0	
Preoperative grade	Grade 1	38 (56.7%)	8 (50.0%)	27 (84.4%)	0.002
	Grade 2	13 (19.4%)	6 (37.5%)	5 (15.6%)	
	Grade 3	16 (23.9%)	1 (6.2%)	0	

Continuous variables are presented as median (IQR), and categorical variables as n (%). Comparisons were performed using the Kruskal–Wallis test, chi-square test, or Fisher’s exact test, as appropriate. Percentages were calculated based on available data. Additional post-hoc comparisons are presented in Supplementary Table S1. TV-USG, transvaginal ultrasonography; PET, positron emission tomography; SUV, standardized uptake value.

Presenting symptoms also varied significantly (*p* < 0.001). Postmenopausal bleeding was the most common symptom in the laparotomy and laparoscopy groups, whereas premenopausal bleeding was more frequently observed in the V-NOTES group. Among imaging findings, endometrial thickness measured by transvaginal ultrasonography was significantly higher in the laparotomy group (*p* = 0.029). In addition, preoperative myometrial invasion differed significantly among groups (*p* = 0.033), with deeper invasion more frequently observed in the laparotomy cohort. Preoperative histopathological characteristics also demonstrated significant differences. Preoperative pathological diagnosis varied across groups (*p* < 0.001), with a higher proportion of high-risk histological subtypes in the laparotomy group. Similarly, preoperative tumor grade differed significantly (*p* = 0.002), with grade 3 tumors being more frequent in the laparotomy group, whereas grade 1 tumors predominated in the V-NOTES group. In contrast, parity did not differ significantly among groups (*p* = 0.071), and no statistically significant differences were observed in smoking status, socioeconomic status, or PET-based parameters (all *p* > 0.05).

### 3.2 Operative characteristics and lymph node assessment

Operative characteristics according to surgical approach are presented in Table 2. There were significant differences in both operation time and estimated blood loss among the groups (both *p* < 0.001). The laparotomy group demonstrated longer operative duration and higher blood loss compared with the minimally invasive groups. The method of lymph node assessment differed markedly among surgical approaches (*p* < 0.001). Extensive lymph node dissection, including bilateral pelvic and para-aortic lymphadenectomy, was predominantly performed in the laparotomy group, whereas SLND was more commonly used in the laparoscopy and V-NOTES groups. Consistent with this, paraaortic lymph node dissection was performed significantly more frequently in the laparotomy group (*p* < 0.001). In addition, the total number of retrieved pelvic lymph nodes differed significantly among groups (*p* = 0.019). When lymph node metastasis was evaluated, positive lymph nodes were identified almost exclusively in the laparotomy group, with only one case observed in the V-NOTES group (*p* < 0.001). Despite these differences in surgical extent, no statistically significant differences were observed in transfusion requirement or intraoperative complication rates among the groups (*p* = 0.475 and *p* = 0.686, respectively).

**Table 2:** Operative characteristics and lymph node assessment.

Variable		Laparotomy *n* = 84	Laparoscopy *n* = 20	V-NOTES *n* = 43	*p*
Operation time, min		110.0 (75.0–120.0)	70.0 (60.0–72.5)	70.0 (65.0–75.0)	<0.001
Estimated blood loss, mL		200.0 (150.0–250.0)	70.0 (50.0–100.0)	80.0 (60.0–100.0)	<0.001
Transfusion requirement		2/83 (2.4%)	0/20 (0%)	0/41 (0%)	0.475
Intraoperative complication		1/84 (1.2%)	0/20 (0%)	0/43 (0%)	0.686
LND method	No LND	0	0	1 (2.7%)	<0.001
	SLND only	19 (23.5%)	10 (58.8%)	32 (86.5%)	
	BPLND	10 (12.3%)	5 (29.4%)	4 (10.8%)	
	BPPLND	52 (64.2%)	2 (11.8%)	0	
Para-aortic dissection performed		46/84 (54.8%)	1/18 (5.6%)	0/40 (0%)	<0.001
Total pelvic LN count		16.0 (12.0–22.0)	18.5 (12.5–22.5)	8.0 (4.0–10.0)	0.019
Right SLN count		2.0 (2.0–4.0)	2.0 (2.0–3.0)	2.0 (2.0–3.8)	0.684
Left SLN count		2.0 (2.0–4.0)	3.0 (2.0–5.0)	2.0 (2.0–4.8)	0.797
Any positive lymph node		23 (27.4%)	0 (0%)	1 (2.3%)	<0.001

Continuous variables are presented as median (IQR), and categorical variables as n (%). Percentages were calculated based on available data. Lymph node–related analyses were performed among patients undergoing the corresponding procedures. Additional post-hoc comparisons are presented in Supplementary Table S1. LND, lymph node dissection; SLND, sentinel lymph node dissection; BPLND, bilateral pelvic lymph node dissection; BPPLND, bilateral pelvic and para-aortic lymph node dissection.

### 3.3 Final pathological characteristics and stage distribution

Final pathological findings and stage distributions are summarized in Table 3. The distribution of final histopathological diagnosis did not reach statistical significance (*p* = 0.066), although numerically, high-risk histological subtypes, including serous carcinoma, carcinosarcoma, and mixed high-risk tumors, were more frequently observed in the laparotomy group. Similarly, postoperative tumor grade (*p* = 0.142) and depth of myometrial invasion (*p* = 0.094) did not differ significantly among groups, although more advanced pathological features were numerically more common in the laparotomy cohort.

**Table 3:** Final pathological characteristics and FIGO stage distribution.

Variable		Laparotomy *n* = 84	Laparoscopy *n* = 20	V-NOTES *n* = 43	*p*
Final pathology	EIN/atypical hyperplasia	0	0	1 (2.3%)	0.066
	Endometrioid carcinoma	58 (69.9%)	15 (83.3%)	36 (83.7%)	
	Serous carcinoma	10 (12.0%)	0	1 (2.3%)	
	Clear cell carcinoma	1 (1.2%)	0	0	
	Sarcoma/carcinosarcoma/mixed high-risk	9 (10.8%)	0	0	
	Normal/no residual malignancy	0	1 (5.6%)	1 (2.3%)	
	Other	5 (6.0%)	2 (11.1%)	4 (9.3%)	
Postoperative grade	Grade 1	26 (36.6%)	8 (50.0%)	19 (55.9%)	0.142
	Grade 2	25 (35.2%)	6 (37.5%)	12 (35.3%)	
	Grade 3	20 (28.2%)	2 (12.5%)	3 (8.8%)	
Myometrial invasion	No invasion	6 (7.5%)	0	3 (8.8%)	0.094
	<50% invasion	24 (30.0%)	7 (46.7%)	19 (55.9%)	
	≥50% invasion	45 (56.2%)	8 (53.3%)	12 (35.3%)	
	Serosal invasion	5 (6.2%)	0	0	
LVSI positivity		22/80 (27.5%)	0/13 (0%)	1/33 (3.0%)	0.002
Cervical stromal invasion		15/80 (18.8%)	1/14 (7.1%)	1/34 (2.9%)	0.058
Uterine serosal involvement		10/81 (12.3%)	0/16 (0%)	0/35 (0%)	0.033
Adnexal involvement		5/80 (6.2%)	0/14 (0%)	0/34 (0%)	0.210
Positive surgical margin		3/67 (4.5%)	0/15 (0%)	0/32 (0%)	0.339
FIGO 2023 stage	IA1	2 (2.5%)	1 (6.7%)	5 (13.9%)	0.010
	IA2	16 (20.0%)	3 (20.0%)	17 (47.2%)	
	IB	21 (26.2%)	8 (53.3%)	11 (30.6%)	
	IC	2 (2.5%)	0	2 (5.6%)	
	IIC	10 (12.5%)	2 (13.3%)	1 (2.8%)	
	IIIA2	2 (2.5%)	0	0	
	IIIB1	1 (1.2%)	0	0	
	IIIB2	2 (2.5%)	0	0	
	IIIC1	8 (10.0%)	0	0	
	IIIC2	7 (8.8%)	0	0	
	IVB	8 (10.0%)	0	0	
			

Continuous variables are presented as median (IQR), and categorical variables as n (%). Percentages were calculated based on available data. FIGO staging analyses were performed among patients with available staging data. Additional post-hoc comparisons are presented in Supplementary Table S1. LVSI, lymphovascular space invasion; FIGO, International Federation of Gynecology and Obstetrics.

In contrast, several adverse pathological features demonstrated significant differences. LVSI positivity was significantly higher in the laparotomy group (*p* = 0.002). Likewise, uterine serosal involvement differed significantly among groups (*p* = 0.033), with all cases occurring in the laparotomy group. Although cervical stromal invasion was more frequently observed in the laparotomy group, this difference did not reach statistical significance (*p* = 0.058). No significant differences were observed for adnexal involvement or positive surgical margins (*p* > 0.05). FIGO 2023 staging demonstrated significant differences among groups (*p* = 0.010). Advanced-stage disease was predominantly concentrated in the laparotomy group, whereas most V-NOTES patients were classified as early-stage disease.

### 3.4 Adjuvant treatment and follow-up outcomes

Adjuvant treatment patterns and follow-up findings are presented in Table 4. The proportion of patients receiving adjuvant treatment differed significantly among groups (*p* < 0.001). Adjuvant therapy was most frequently administered in the laparotomy group and least frequently in the V-NOTES group. Among patients who received treatment, the distribution of treatment modalities also differed significantly (*p* = 0.021). Combined treatment strategies, including chemoradiotherapy and multimodal treatment approaches, were more commonly used in the laparotomy group, whereas brachytherapy alone was more frequently observed in the minimally invasive groups. Follow-up duration could be calculated in 87 patients using the interval between diagnosis date and the last available follow-up visit. Median follow-up duration did not differ significantly among groups (*p* = 0.570). No recurrences were observed in the laparoscopy or V-NOTES groups. During follow-up, only one recurrence event was documented, occurring in the laparotomy group. Similarly, PET findings suspicious for recurrence, ultrasonographic abnormalities during follow-up, and tumor marker levels (CA-125 and CEA) did not differ significantly among groups (all *p* > 0.05). Formal disease-free survival and overall survival analyses could not be performed because recurrence-date and survival-event data were not consistently available in the dataset.

**Table 4:** Adjuvant treatment patterns and follow-up findings according to surgical approach.

Variable	Laparotomy *n* = 84	Laparoscopy *n* = 20	V-NOTES *n* = 43	*p*
Adjuvant treatment administered*	54/81 (66.7%)	10/19 (52.6%)	11/42 (26.2%)	<0.001
Adjuvant treatment type among treated patients				0.021
Chemotherapy only	3 (5.7%)	0	0	
Radiotherapy only	0	1 (10.0%)	0	
Chemoradiotherapy	7 (13.2%)	0	1 (9.1%)	
Brachytherapy only	19 (35.8%)	7 (70.0%)	9 (81.8%)	
Chemoradiotherapy + brachytherapy	8 (15.1%)	2 (20.0%)	1 (9.1%)	
Chemoradiotherapy + external abdominal RT	16 (30.2%)	0	0	
Patients with calculable follow-up duration	51	12	24	—
Follow-up duration, months	12.1 (7.2–15.2)	13.6 (11.3–15.6)	12.7 (8.7–15.4)	0.570
Follow-up duration range, months	0.7–27.5	5.2–18.8	2.5–23.1	—
Recurrence among followed patients	1/54 (1.9%)	0/11 (0%)	0/26 (0%)	—
PET finding suspicious for recurrence†	3/39 (7.7%)	0/8 (0%)	1/14 (7.1%)	0.722
Abnormal follow-up USG finding	1/49 (2.0%)	0/9 (0%)	1/24 (4.2%)	0.756
Follow-up CA-125, U/mL	9.1 (7.0–12.3)	10.1 (7.2–12.8)	10.0 (6.5–20.0)	0.923
Follow-up CEA, ng/mL	2.1 (1.8–3.0)	1.1 (0.9–2.0)	2.0 (1.8–2.5)	0.108

*Treatment refusal was excluded from binary adjuvant treatment comparisons. †PET variable was coded as 0 = suspicious recurrence finding and 1 = no suspicious recurrence finding. Continuous variables are presented as median (IQR), and categorical variables as n/N (%). Follow-up analyses were limited to patients with available follow-up data. Formal survival analyses were not performed because recurrence-date and survival-event data were inconsistently available. PET, positron emission tomography; USG, ultrasonography; CEA, carcinoembryonic antigen; RT, radiotherapy.

### 3.5 Multivariable analysis of factors associated with adjuvant treatment

A multivariable logistic regression analysis was performed to identify factors associated with adjuvant treatment requirement (Table 5). After adjustment for potential confounders, postoperative tumor grade (OR = 5.78, 95% CI: 2.29–14.63, *p* < 0.001) and deep or serosal myometrial invasion (OR = 35.23, 95% CI: 7.78–159.50, *p* < 0.001) remained independent predictors of adjuvant treatment. In contrast, surgical approach was not independently associated with adjuvant treatment requirement. Neither laparoscopy (OR = 1.89, *p* = 0.518) nor V-NOTES (OR = 0.26, *p* = 0.068) demonstrated a statistically significant association after adjustment. Age and LVSI status were also not independently associated with adjuvant treatment in the multivariable model (*p* > 0.05).

**Table 5:** Multivariable logistic regression for adjuvant treatment requirement.

Variable	OR	95% CI	*p*
Age	1.03	0.96–1.09	0.414
Laparoscopy vs. laparotomy	1.89	0.27–13.11	0.518
V-NOTES vs. laparotomy	0.26	0.06–1.10	0.068
Postoperative grade	5.78	2.29–14.63	<0.001
Deep/serosal myometrial invasion	35.23	7.78–159.50	<0.001
LVSI positivity	1.07	0.09–12.18	0.956

Model *n* = 110. Outcome: adjuvant treatment. Multivariable logistic regression analysis was performed to identify factors associated with adjuvant treatment requirement. Variables included in the model were selected based on clinical relevance and univariate analysis results. The model included age, surgical approach, postoperative tumor grade, myometrial invasion depth, and LVSI status. Results are presented as odds ratios (OR) with 95% confidence intervals (CI). Only cases with complete data for all variables included in the model were analyzed. Patients who refused treatment and those with missing data were excluded from the regression analysis. OR, odds ratio; CI, confidence interval; LVSI, lymphovascular space invasion.

### 3.6 Adjusted analysis of perioperative outcomes according to surgical approach and lymph node assessment

To further evaluate whether perioperative differences were independently associated with surgical approach or influenced by the extent of lymph node assessment, adjusted linear regression analyses were performed for operative time and estimated blood loss (Table 6). Surgical approach and lymph node dissection (LND) type were included simultaneously in the regression models. After adjustment for LND type, both laparoscopy and V-NOTES remained independently associated with shorter operative time compared with laparotomy (*p* = 0.020 and *p* = 0.006, respectively). Similarly, estimated blood loss remained significantly lower in the minimally invasive groups after adjustment (both *p* < 0.001). In contrast, systematic lymph node dissection was independently associated with longer operative duration and higher estimated blood loss compared with SLND alone (both *p* < 0.001). These findings suggest that perioperative differences among surgical groups were influenced not only by surgical approach itself, but also by the extent and complexity of lymph node assessment procedures.

**Table 6:** Adjusted linear regression analysis for operative time and estimated blood loss according to surgical approach and lymph node assessment strategy.

Variable	Operative time, B (95% CI)	*p*	Estimated blood loss, B (95% CI)	*p*
Laparoscopy vs. laparotomy	−16.27 (−29.98 to −2.56)	0.020	−88.49 (−133.56 to −43.41)	<0.001
V-NOTES vs. laparotomy	−13.16 (−22.51 to −3.82)	0.006	−88.51 (−118.17 to −58.86)	<0.001
Systematic LND vs. SLND only	26.48 (16.67 to 36.29)	<0.001	55.51 (22.99 to 88.02)	<0.001

*B* values represent adjusted unstandardized regression coefficients. Surgical approach and lymph node dissection type were included in the same model. Laparotomy and SLND only were used as reference categories. Systematic LND included bilateral pelvic lymph node dissection and bilateral pelvic plus para-aortic lymph node dissection. Cases with missing LND method or no lymph node dissection were excluded from this model. Robust standard errors were used.

## 4 Discussion

The management of endometrial cancer has undergone a substantial shift over the past two decades, with minimally invasive approaches increasingly replacing laparotomy in appropriately selected patients (31). This transition has been largely driven by consistent evidence demonstrating reduced perioperative morbidity without compromising oncologic outcomes in early-stage disease. However, the interpretation of surgical outcomes in real-world cohorts remains challenging due to the strong influence of clinical selection bias, which is inherent to observational surgical studies (32, 33).

In this context, the present study should not be viewed as a direct comparison of surgical techniques, but rather as a reflection of how different surgical approaches are applied across heterogeneous patient populations in routine clinical practice. The observed distribution of baseline characteristics appears to support this interpretation. Patients undergoing laparotomy in this cohort exhibited features traditionally associated with higher oncologic risk, including older age, postmenopausal status, deeper myometrial invasion, higher tumor grade, and a greater prevalence of non-endometrioid histology. Among baseline variables, the marked differences in preoperative pathology and preoperative tumor grade likely represent one of the strongest indicators of non-random surgical allocation within the cohort and may have substantially influenced subsequent perioperative, pathological, and treatment-related findings. In contrast, V-NOTES was predominantly performed in younger patients with low-grade, uterine-confined disease. This pattern is consistent with established surgical decision-making algorithms, where the anticipated need for extensive staging and the likelihood of extrauterine disease guide the choice of surgical approach (28, 34).

Within this framework, the perioperative findings of the study become more interpretable. The longer operative times and increased blood loss observed in the laparotomy group may not necessarily reflect an inherent limitation of open surgery, but rather the greater complexity of the surgical procedures performed in this group, including more frequent para-aortic dissection and comprehensive lymphadenectomy. Conversely, the shorter operative duration and lower blood loss in the minimally invasive groups likely reflect the combination of lower tumor burden and less extensive surgical staging, rather than a purely technique-related advantage. This distinction is critical, as failure to account for procedural complexity can lead to misleading conclusions regarding surgical performance.

An additional layer of complexity arises from differences in lymph node assessment strategies. The increasing adoption of sentinel lymph node mapping has significantly altered the surgical landscape of endometrial cancer (35, 36). In the present cohort, SLND was predominantly utilized in minimally invasive approaches, whereas systematic pelvic and para-aortic lymphadenectomy was concentrated in the laparotomy group. Given the well-documented association between extensive lymphadenectomy and increased operative time, blood loss, and postoperative morbidity, the observed perioperative differences between groups may have been influenced not only by surgical approach, but also by concurrent evolution in staging strategies (37, 38). Therefore, surgical route and lymph node assessment method should be considered as interrelated variables rather than independent factors.

The distribution of pathological findings appears to be consistent with the influence of baseline risk stratification and non-random surgical allocation across surgical groups. Although the overall distribution of histological subtypes did not reach statistical significance, adverse features such as LVSI positivity, serosal involvement, and advanced-stage disease were clearly concentrated in the laparotomy group. This clustering of high-risk characteristics may be expected in routine clinical practice and reflects the tendency to reserve open surgery for patients with suspected extrauterine spread or aggressive tumor biology (39). Accordingly, the differences in adjuvant treatment rates observed across the groups are likely a downstream consequence of this risk stratification process (40, 41).

This interpretation is further supported by the multivariable analysis. After adjusting for key pathological factors, surgical approach was not independently associated with adjuvant treatment requirement, whereas postoperative tumor grade and depth of myometrial invasion remained significant predictors. These findings highlight that treatment decisions appear to be more closely associated with tumor biology than surgical modality, and that unadjusted comparisons between surgical groups may overestimate the apparent effect of surgical approach (42, 43).

The evaluation of oncologic follow-up findings in this study is limited by the extremely low number of recurrence events, the relatively short follow-up duration, and the absence of consistently recorded recurrence-date and survival-event data. Although no recurrences were observed in the minimally invasive groups, this finding should not be interpreted as evidence of oncologic equivalence or superiority. Rather, it likely reflects the favorable baseline risk profile of patients selected for minimally invasive approaches together with insufficient follow-up to capture late recurrences. This limitation is particularly important because recurrence is a time-dependent outcome that requires adequately powered cohorts and standardized long-term follow-up for meaningful interpretation.

When considered in the context of existing literature, the findings of this study are not contradictory but rather complementary. Randomized controlled trials such as LAP2 and LACE have demonstrated that minimally invasive surgery provides comparable oncologic outcomes to laparotomy in early-stage endometrial cancer, with clear perioperative advantages (44–46). However, these trials were conducted under controlled conditions with predefined eligibility criteria. In contrast, the present study reflects real-world practice, where patient selection is inherently non-random and influenced by multiple clinical factors. As such, the current findings highlight the importance of distinguishing between efficacy under controlled conditions and observations derived from routine clinical practice.

Several limitations should be acknowledged when interpreting the present findings. First, the retrospective design introduces the possibility of selection bias and residual confounding. The marked imbalance in baseline clinicopathological characteristics between surgical groups limits causal interpretation of the observed associations. In particular, the relatively small size of the laparoscopy subgroup reduced statistical power for certain between-group comparisons and may have limited the ability to detect modest differences between minimally invasive approaches. Accordingly, comparisons involving the laparoscopy cohort should be interpreted cautiously. Second, although follow-up duration data were available for a subset of patients, the relatively short and incomplete follow-up together with the absence of consistently recorded recurrence-date and survival-event information precluded meaningful survival analyses. In addition, the very low number of recurrence events limited interpretation of oncologic follow-up findings. Missing data for several pathological and follow-up variables may also have introduced additional bias, particularly if missingness was not completely random. Third, surgical approach selection was influenced by institutional practice patterns, surgeon preference, and anticipated procedural complexity in routine clinical practice. Procedures were performed by multiple gynecologic oncologists rather than a single operator, and detailed surgeon-level data were not consistently available. Therefore, inter-operator variability and differences in surgical experience may have influenced perioperative findings and represent additional sources of residual confounding. Furthermore, para-aortic lymph node dissection was not routinely performed through the V-NOTES approach because of technical and patient-selection considerations, which may have further contributed to differences in surgical staging patterns between groups.

## 5 Conclusion

The present study suggests that differences in perioperative outcomes, pathological characteristics, and adjuvant treatment patterns among laparotomy, laparoscopy, and V-NOTES are likely associated with underlying variations in patient and tumor profiles, rather than the intrinsic effects of the surgical approaches themselves. In routine clinical practice, minimally invasive techniques appeared to be preferentially applied in patients with lower-risk, uterine-confined disease, whereas laparotomy remained more frequently utilized in patients with advanced-stage or high-risk tumors requiring more extensive surgical staging. Accordingly, the apparent advantages observed in minimally invasive approaches should be interpreted within the context of patient selection and procedural complexity. The findings further suggest that tumor-related factors, particularly postoperative grade and depth of myometrial invasion, appeared to be more strongly associated with adjuvant treatment requirement than the choice of surgical approach. Given the retrospective design, baseline imbalances, and limited follow-up data, this study should be considered a descriptive analysis of real-world surgical practice rather than a comparative effectiveness evaluation. Future research incorporating prospective designs, standardized selection criteria, and advanced statistical adjustment methods is required to better delineate the independent impact of surgical approach on oncologic outcomes.

## Data Availability

The raw data supporting the conclusions of this article will be made available by the authors, without undue reservation.
