# Supplementary material for: Surgical approach selection and perioperative-pathological characteristics in endometrial cancer: a retrospective observational study
Source: Front Med (Lausanne). 2026 Jun 17;13:1870788. doi: 10.3389/fmed.2026.1870788 (PMC13319022; doi:10.3389/fmed.2026.1870788)
Supplement: Supplementary file 1 [file Data_Sheet_1.PDF]

Supplementary Table S1. Post-hoc pairwise comparisons between surgical groups for clinically relevant perioperative and clinicopathological variables

Variable	Comparison	Adjusted p
Menopausal status	Laparotomy vs Laparoscopy	1.000
	Laparotomy vs V-NOTES	0.011
	Laparoscopy vs V-NOTES	0.618
Preoperative grade	Laparotomy vs Laparoscopy	1.000
	Laparotomy vs V-NOTES	0.002
	Laparoscopy vs V-NOTES	0.071
LND method	Laparotomy vs Laparoscopy	<0.001
	Laparotomy vs V-NOTES	<0.001
	Laparoscopy vs V-NOTES	0.018
Para-aortic dissection	Laparotomy vs Laparoscopy	<0.001
	Laparotomy vs V-NOTES	<0.001
	Laparoscopy vs V-NOTES	0.371
LVSI positivity	Laparotomy vs Laparoscopy	0.041
	Laparotomy vs V-NOTES	0.003
	Laparoscopy vs V-NOTES	1.000
FIGO stage	Laparotomy vs Laparoscopy	0.214
	Laparotomy vs V-NOTES	0.004
	Laparoscopy vs V-NOTES	0.537
Adjuvant treatment	Laparotomy vs Laparoscopy	0.804
	Laparotomy vs V-NOTES	<0.001
	Laparoscopy vs V-NOTES	0.148

Pairwise comparisons were performed only for variables demonstrating significant overall group differences in the primary analyses. Bonferroni-adjusted Mann–Whitney U tests were used for continuous variables, and Bonferroni-adjusted chi-square/Fisher’s exact tests were used for categorical variables. Adjusted p-values are presented. Significant p-values are shown in bold.

Supplementary Table S2. Missing data summary

Variable	Available n	Missing n (%)
LVSI status	126	21 (14.3%)
Adjuvant treatment	142	5 (3.4%)
Follow-up duration	87	60 (40.8%)
Recurrence status	91	56 (38.1%)
Follow-up CA-125	65	86 (57.0%)
Follow-up CEA	65	86 (57.0%)
Logistic regression complete cases	110	37 (25.2%)
